# Society for Cardiovascular Magnetic Resonance guidelines for reporting cardiovascular magnetic resonance examinations

**DOI:** 10.1186/1532-429X-11-5

**Published:** 2009-03-03

**Authors:** W Gregory Hundley, David Bluemke, Jan G Bogaert, Matthias G Friedrich, Charles B Higgins, Mark A Lawson, Michael V McConnell, Subha V Raman, Albert C van Rossum, Scott Flamm, Christopher M Kramer, Eike Nagel, Stefan Neubauer

**Affiliations:** 1 Department of Internal Medicine, Section on Cardiology and Radiology, Wake Forest University School of Medicine, Winston-Salem, USA; 2 Radiology and Imaging Sciences National Institutes of Health, Bethesda, USA; 3 Department of Radiology, Medical Imaging Research Center, Leuven, Belgium; 4 Department of Cardiac Sciences & Radiology, University of Calgary, Calgary, Canada; 5 Department of Radiology, UCSF Medical Center, San Francisco, USA; 6 Division of Cardiovascular Medicine, Vanderbilt University School of Medicine, Nashville, USA; 7 Cardiovascular Medicine, Stanford School of Medicine, Stanford, USA; 8 Department of Internal Medicine/Cardiovascular Medicine, The Ohio State University, Columbus, USA; 9 Department of Cardiology, VU University Medical Center, Amsterdam, the Netherlands; 10 Imaging, Heart and Vascular Institutes, Cleveland, USA; 11 Department of Medicine and Radiology, University of Virginia Health System, Charlottesville, USA; 12 Division of Imaging Sciences, King's College London BHF Centre, NIHR Biomedical Research Centre at Guy's & St. Thomas', NHR Foundation Trust, London, UK; 13 Department of Cardiovascular Medicine, University of Oxford, Oxford, UK

## Abstract

These reporting guidelines are recommended by the Society for Cardiovascular Magnetic Resonance (SCMR) to provide a framework for healthcare delivery systems to disseminate cardiac and vascular imaging findings related to the performance of cardiovascular magnetic resonance (CMR) examinations.

## Background

These reporting guidelines are recommended by the Society for Cardiovascular Magnetic Resonance (SCMR) to provide a framework for reporting results of cardiovascular magnetic resonance (CMR) examinations. This document builds on previously published guidelines from professional societies (ACC/AHA/ACR and others) [1], and is customized here for CMR practice in particular. The guidelines have been developed within the context of the US health-care system, and application in other health-care systems may vary. It is also recognized that the ultimate judgment regarding the propriety of any specific procedure or reporting methodology must be made by the physician or individuals participating within the healthcare delivery system that performs the CMR procedure. An alternative approach that differs from these guidelines, standing alone, does not necessarily imply that the different approach falls below the standard of care. To the contrary, a conscientious practitioner may reasonably adopt reporting elements different from those set forth in these recommendations when, in the reasonable judgment of the practitioner, such course of action is indicated by the condition of the patient, limitations of available resources, or a new advancement in knowledge or technology that may occur subsequent to the publication of this document.

Prior to scanning, the SCMR recommends that patients be referred for CMR scans in accordance with Appropriateness Criteria developed by the SCMR, ACC, ACR, and AHA [2]. The SCMR recommends that scans should be performed in accordance with SCMR developed Guidelines for scan acquisition [3].

The SCMR recommends reporting key elements in all documents including information pertaining to a) site and equipment information, b) patient demographics, c) indications for study, d) study performance, e) cardiovascular imaging features of the examination, and f) concluding statements that synthesize the study results into a comprehensive diagnosis that can be used for planning therapy or determining prognosis.

The SCMR wishes to emphasize that effective communication is an essential component of any diagnostic imaging procedures for patients with possible cardiovascular disease. Quality patient care is best achieved when study results are conveyed in a timely fashion to those ultimately responsible for treatment decisions. Accordingly, the SCMR recommends that a delivered, finalized report be available, where possible, within 1 business day of performance of the scan, but appropriate to the urgency of the examination.

The following document serves as a guide to identify a) recommended and optional components of the report, b) the principles used to generate a final report, and c) suggested communications that may occur other than the final report. A final written interpretation or report shall be generated and archived following any CMR examination, procedure, or officially requested consultation to review images regardless of the setting where the CMR scan was performed (hospital, imaging center, physician office, mobile unit, etc.). Within the document, all recommended and optional components are in bold, and summarized in Additional files 1 and 2.

## Components of the report

1) Administrative – 5 total elements (3 recommended; 2 optional)

a. **Site ID** (recommended): Site ID is a unique number assigned to each study performance site.

 b. **Site of service** (recommended): Indicate the type of facility submitting the reporting data. These would include inpatient hospital, outpatient facility, free standing imaging center, ambulatory care office, or mobile unit.

 c. **Scanner** (recommended): Indicate the type of magnet, manufacturer, model number, field strength, and software platform of the unit performing the procedure.

 d. **Accreditation status** (recommended): This should be represented as yes, pending, or no.

 e. **Accreditation Entity** (recommended): For example, (i.e., Intersocietal Committee for Accreditation of Magnetic Resonance Laboratories, etc.).

2) Demographics (4 recommended elements)

 a) **Unique patient ID**: medical record number used by the health care delivery system where the CMR examination was performed

 b) **Patient date of birth**

 c) **Patient Gender **

d) **Patient Race/Ethnicity**

3) Study Referral Data (2 optional elements)

 a) **Referral physician - National Provider Identifier** (NPI)

b) **Referral physician specialty**

4) Scheduling and Performance of Study (6 recommended elements)

a) **Date of procedure**

b) **Time of procedure**

c) **Personnel involved in procedure**

• Nursing

• House Officers

• Staff physicians

• Technologists

d) **Primary indication for test**

e) **Study quality**

f) **Listing of sequences used**

• T_1 _W dark blood with or without contrast

• T_2 _W dark blood with and/or without fat saturation

• Cine SSFP

• Cine FGRE

• Late gadolinium enhancement

• Phase contrast CMR flow measurements

• T_1 _W MRA with or without contrast

• T2*

• SPAMM (Grid Tagging)

5) History and Risk Factors (2 recommended and 1 optional element)

a) **Height** (recommended)

b) **Weight** (recommended)

c) For studies using contrast, the SCMR suggests the value and date of acquisition of the most recent **serum creatinine level **and estimated **glomerular filtration rate **(GFR) should be provided.

6) Non-imaging findings associated with the examination (5 recommended elements)

a) In those studies requiring 12-lead **electrocardiogram**, its **interpretation **should be provided. This includes the presence of Q-waves, ST segment or T-wave abnormalities, or other rhythm disturbances.

b) For studies evaluating hemodynamically important conditions (i.e., valvular heart disease, intracardiac shunting, cardiac output, etc.), **heart rates and rhythm**, and **systolic **and **diastolic blood pressure **should be provided during the CMR acquisition. For tests incorporating stress testing, the heart rates and rhythm, **oxygen saturation**, systolic and diastolic blood pressures, and the **predictive heart rate response for age **should all be recorded during the following points in time:

• Before study

• At each level of stress

• In recovery

c) For studies utilizing cardiac active agents (i.e., stress testing), the **agent**, **quantity**, **duration**, and **route of administration of the agents and associated medications **should be provided.

d) For studies utilizing contrast **agents, the type (i.e. paramagnetic), name, route, site, and speed of administration **should be provided.

e) For studies utilizing sedation, general anesthesia, or supported ventilatory or cardiac (hemodynamic or electrical) assistance, the **amount, type, route and measures of administration of these agents or support **should be documented. Also, patients' **cardiovascular and pulmonary responses **(heart rate, blood pressure, respiratory rate, and oxygen saturation) should be recorded accordingly to local regulations. The **reason for administration **required of the agent should be provided.

7. Specific Conditions Assessed with CMR

a) Magnetic resonance arteriography

1. Aorta

Dimensions including (4 recommended, 1 optional):

a. **Aortic annulus** (recommended)

b. **Sinuses of Valsalva** (recommended)

c. **Sinotubular junction** (recommended)

d. **Ascending and descending diameters** at the level of the pulmonary artery (recommended)

e. Comment regarding whether the aorta is right or left-sided may be provided (optional).

Findings when present (7 recommended, 1 optional):

a. Comment on **sinotubular effacement** (recommended)

b. Comment on **tortuosity** (recommended)

 c. **Aortic atherosclerosis** (recommended): description of location, mobility and extent, estimate %-stenosis when advanced

 d. **Aortic aneurysm** (recommended): **size **(AP × LR × CC), **morphology **(saccular versus fusiform), **location **in the aorta, **relation to branch vessels**, **presence of mural thrombus**, **visceral compressive effects (effacement expansion of the aorta against surrounding structures)**, **post-contrast appearance **(if these sequence were acquired), presence of **periaortic**, **mediastinal**, **pericardial**, or **pleural fluid**.

 e. **Aortic dissection** (recommended): **dissection classification **(either DeBakey or Stanford), presence of **intimal flap**, **location of tear or areas of communication **(if possible), description of the **size and extent of the true and false lumens**, presence of **murmal thrombus or blood in false lumen**, **branch vessel involvement**, **presence of periaortic, mediastinal, pericardial, or pleural fluid**,

i. **Intramural hematoma** (IH): in cases of IH and **penetrating aortic ulcer**, the CMR practitioner should describe carefully the morphologic findings in much the same way as an aortic dissection paying careful attention to select wording to convey a diagnosis of limited ulceration or dissection.

ii. Post-operative appearance: this should be described in accordance with (a-e) above noting additional graft insertion points and dimensions.

 f. **Inflammatory diseases** of the aorta (recommended): **aortic wall thickness**, **multispectral appearance on different pulse sequences**, **contrast enhancement pattern**, **branch vessel involvement**, **presence of periaortic, pleural, or pericardial fluid**

g. Congenital disease involving the aorta and ventriculoarterial connections: see recommended congenital report below.

 h. **Aortic flow** (optional): On CMR scans of the aorta in which PC-MR measures are obtained, the direction and magnitude of flow should be provided.

2. Peripheral arterial disease (2 recommended, 1 optional):

a) **Vessel location and orientation**. Descriptions of each territory are required when the study is ordered to examine the respective site (recommended). When severe stenoses or vessel occlusions are identified, common collateral pathways should be described.

• Arch vessels

• Carotid bifurcation

• Celiac trunk

• Proximal SMA

• Renal arteries and their accessory vessels

• Common and external iliac

• Femoral, brachial or other more peripheral arteries.

b) Quantitation of luminal narrowings or stenoses (recommended)

SCMR recommends that the CMR practitioner avoid descriptive terms such as "mild" or "moderate" stenosis, but rather adopt a semi-quantitative method that scores the severity of luminal occlusion. Accordingly, **stenosis severity should be reported in 25% increments **(i.e., <25%, 26% – 50%, 51% – 75%, and >75%) or, in cases with high spatial resolution, finer increments of 10% may be employed. Descriptive terms may convey the wrong impression to the clinical importance of occlusive disease (e.g. a series of "moderate" stenoses in the diabetic patient with poor wound healing of the lower extremity may be clinically significant)

c) Optional functional measures of the vascular system may also be reported, including:

i.) **flow measurements** in the forms of milliliters or liters per minute, and

ii.) **measures of vascular stiffness**: aortic distensibility, or pulse wave velocity.

When functional measures are provided it is recommended that the vascular territories be specified and values provided at the specific location of acquisition.

b) Cardiac Size and Function

1) The reporting of **right ventricular (RV) **and **left and right atrial chamber sizes **and **volumes **are optional. Reporting of **left ventricular (LV) volumes **is recommended when multi-slice cine short axes data are acquired from the mitral annulus to the cardiac apex. When reported, SCMR suggests that right-sided chambers measurements and the angulation from which the diameters or dimensions are acquired should be reported. For the left-sided cardiac chambers, the 3-chamber long axis view should be used for identifying **LV dimensions**. The SCMR encourages quantitative measures reported on their forms; however, determination of normal, enlarged, small, or not reported may be substituted.

2) Although not required, the SCMR encourages the reporting of **LV end diastolic wall thicknesses **acquired in the 3-chamber view of the left ventricle at the mitral leaflet tips; it is suggested that the end diastolic thickness be acquired at the septum and inferior lateral or posterior wall.

3) When assessing the **right ventricle**, **the free wall end diastolic thickness **(in the middle atrium portion of the wall) may be reported.

4) For those studies targeting the heart, the SCMR recommends the reporting of **LV ejection fraction, and regional wall motion abnormalities**. The method of acquisition should be reported, including:

▪ Visual estimation

▪ Area-length formula

▪ Multi-slice disk summation technique

Values should be reported as absolute values and indexed for body surface area.

**Figure 1:**
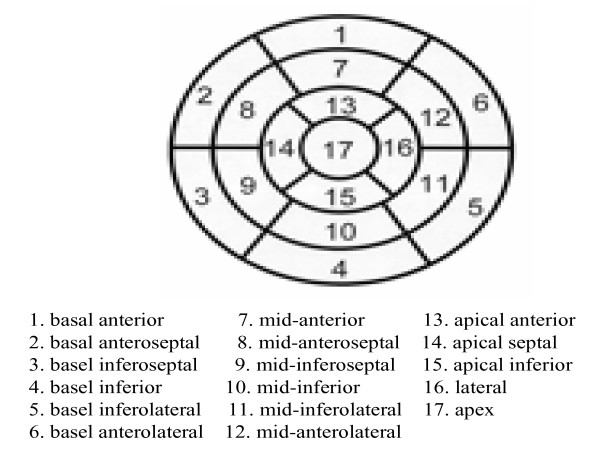
**17-segment model of the LV myocardium developed by the ACC/AHA for reporting abnormalities of wall motion, perfusion or injury**.

• Measurements derived from these values (i.e., cardiac output) should be expressed as absolute as well as indexed values and the reference heart rates used for these calculations should be provided in the report.

• Regional wall motion should be described as qualitatively or quantitatively assessed in the 17-segment model adopted by the ACC/AHA guidelines^2 ^for noninvasive testing (Figure 1). Qualitative assessments should follow the following nomenclature in which each segment is identified as:

▪ hyperkinetic

▪ normokinetic

▪ hypokinetic

▪ akinetic

▪ dyskinetic

▪ tardykinetic

▪ paradoxical

▪ not assessed

• If the respective site seeks to report quantitative measures, such as thickening or strain, these should be performed and reported according to previously published techniques.

c) **Cardiovascular stress testing**

As described in the non-imaging findings component of the reported list above, parameters such as vital signs, medications, and contrast agent administration should be reported. The SCMR recommends the reporting of LV myocardial information in the format of a 17-segment model through the use of a chart, table, or bipolar maps (so called **"Bullseye" plot**) [4].

1. Wall motion stress:

**Wall function **should be designated as qualitative (wall motion) or quantitative (referenced measure such as % wall thickening, or strain) during testing. In addition, **wall motion score index **(the sum of the wall motion scores divided by the number of segments scored) should be reported at each level of stress. Inducible ischemia or contractile reserve should be identified in each study according to previously published referenced methods. Identification should be made of when global LV function does not improve or worsens during stress.

2. Gadolinium, 1^st ^pass myocardial perfusion:

Existing literature regarding the prognostic significance of qualitative perfusion defects is unavailable at this time; nevertheless, SCMR suggests that perfusion in each of the 17 segments (Figure 1) be defined according to the **transmurality**, and **persistence **of the defect. The committee recommends that stress induced (vasodilator or inotropic) perfusion defects be compared with co-registered rest perfusion or late enhancement segments in order to identify ischemic, infarcted, or non-ischemic areas. The SCMR also recognizes that observed defects may be characterized as artifacts. These should be described.

3. **Late gadolinium enhancement** (LGE):

The amount of intense signal >2 SD above the average of normal myocardium should be reported for the area within each segment. Overall, LGE should be described as subepicardial, intramural, subendocardial, or transmural. Patchy or linear streaks of LGE should be identified. The transmural extent of the LGE should be defined as 0, ≤ 25%, 26% to ≤ 50%, 51% to ≤ 75%, and 76% to 100%. In addition, the total amount of infarcted tissue (volume or grams) relative to the total myocardial volume or mass (g) may be reported. It is not recommended, but measures of LV end-diastolic wall thickness for the 17 myocardial segments may also be reported. When clinically appropriate, those providing an interpretation should indicate whether the pattern of LGE is consistent with ischemic heart disease, myocarditis, etc.

4. **Microvascular obstruction** (MVO) :

If MVO is observed during LGE, its location and presence within the 17 myocardial segments should be provided.

Integrative stress imaging:

It is recognized by the SCMR that the procedures mentioned above can be performed in a single setting and thus must be integrated to arrive at a diagnosis. The committee recommends reporting data for all 17 myocardial segments in all modalities (Figure below). Based on previously published techniques, segments should be identified as ischemic, infarcted, mixed ischemia/infarction, or normal. It is recommended that all information for baseline function be reported in patients referred for stress testing or evaluation of acute or chronic ischemic syndromes.

For these clinical conditions, the following items are recommended for reporting by the SCMR.

• **LV volumes** (EDV, ESV, SV, EF) with and without indexing to body surface area.

• **Presence and extent of (T2) signal intensity**

• **Presence and extent of irreversible injury** (LGE)

• **Presence of pericardial effusion**

• In the case of iron overload: T2* in ms may be reported

• Optional: **early enhancement ratio**† or the % injury related to LV mass

†: see Lake Louise Criteria, Consensus Group on CMR in Myocarditis

e. **Coronary Arterial Segments**

It is recommended that when examining the course of anomalous coronary arteries, the **origin **and **course of the coronary artery segments **be reported, as well as the **length of the segments **visualized. If anomalous artery is **intramural**, it should be noted. If a study is performed for the purpose of identifying coronary artery or bypass graft anatomy, the **patency **of these conduits should be indicated.

f. Valvular Heart Disease

The following lists of items should be reported for the cardiac valves.

Qualitative parameters:

• **morphology **of each component of the valve complex (e.g. leaflets, annulus, chordae)

• presence of any **insufficiency **or reduced **valvular excursion**

When quantitative flow measurements are acquired:

• the velocity encoding **Venc setting**;

• the **peak velocity**, a single value when recorded across semilunar valves or a vessel in cross-section, or both early (E) and late (A) peak velocities for atrioventricular valves;

• the forward stroke volume and peak and mean **transvalvular gradients**;

• the **regurgitant volume and fraction**;

• the **heart rate **during acquisition;

• the method and determination of **valve area **(by planimetry or the continuity equation);

• the measurement of **ventricular dimensions and volumes **as described in III-B.

g. Arrhythmogenic RV Cardiomyopathy (ARVC)

1) It is recommended that each report identify major and minor criteria associated with ARVC. This should include a statement regarding:

a) **Global right ventricular performance **(RVEF);

b) **RV dilation**;

c) Location of **Regional RV wall motion **abnormalities (infundibulum, body or apex of right ventricle).

When acquired:

a) **Fatty infiltration of the right ventricle**, and

b) Occurrence of **fibrosis by LGE **should be provided.

h. Cardiac and paracardiac masses (including pericardium)

The standard report should consist of the following components:

Myocardial mass description:

Absent

Present

Location (pericardial, myocardial, valve relationship, chamber relationship)

Size (cross-sectional dimensions)

T1 signal intensity (homogeneous, heterogeneous, hyper, iso or hypo intense to myocardium/or chest wall (specify reference tissue)

T1 fat sat images signal intensity (if performed) (homogeneous, heterogeneous, hyper, iso or hypo intense to myocardium/or chest wall (specify reference tissue)

T2 signal intensity (homogeneous, heterogeneous, hyper, iso or hypo intense to myocardium/or chest wall (specify reference tissue)

STIR signal intensity

Perfusion pattern (if perfusion performed)

Late gadolinium enhancement pattern on static/delayed images (if gadolinium administered)

Relationship to myocardium/pericardium, mediastinum

Margins (e.g., smooth, irregular, infiltrating, pediculated)

Cine CMR appearance (pedunculated, motion with myocardium/pericardium)

** Myocardial function** (if performed, qualitative or quantitative as appropriate)

** Pericardial abnormalities** if present (pericardial thickness should be reported along with determination of the presence or absence of a pericardial effusion)

Pericardial description

** Morphology** (descriptive)

1. Pericardial Thickness: describe as local or circumferential and list thickness measurements

2. Pericardial effusion (None, trace, small, moderate, large)

 Ventricular parameters

1. **LV volumes **(EDV, ESV, SV, **EF**) with and without indexing for body surface area.

Ventricular **wall motion**

1. **Systolic wall motion**

2. **+/- abnormal septal motion **during normal respiration and breath holding.

4. Presence or absence of **atrial inversion**

 Late Gadolinium Enhancement

1. RV – site

2. LV – site

3. Pericardium

i. Pulmonary Vein assessments

Qualitative elements that should be included in CMR-based PV reporting include:

1. **Number **of pulmonary veins;

2. **Atrial side of pulmonary vein return**;

3. **Recongition of accessory or anomalous pulmonary veins**; and

4. Presence or absence of **stenosis in each PV**, especially in reporting post-ablation CMR exams.

Quantitative elements that should be included in CMR-based PV reporting are:

1. **Maximum ostial diameter** of each pulmonary vein;

2. **Cardiac phase** (e.g. end-atrial diastole) and respiratory phase (e.g. end-expiration) during acquisition of images used for ostial measurements;

3. **Minimum ostial diameter** of each stenotic pulmonary vein; and

4. **Imaging technique** used for measurements

The number and position of pulmonary veins is accounted for noting common trunks, accessory veins, and evidence for stenosis or thrombosis cross sectional area of the pulmonary vein may be provided. A 3D workstation may be used to calculate major and minor axes, and cross sectional area of each pulmonary vein ostium, and compare pre- and post-ablation images side by side.

SCMR recognizes the value of pictorial display of the pulmonary vein orientations, and suggests implementation of diagrams when feasible.

j. Congenital Heart Disease

Morphology (descriptive)

a. Simple Lesions

b. Complex Lesions

i. **situs**

ii. **ventriculoarterial relationship**

iii. **artioventricular relationship**

iv. **pulmonary venous connection**

v. **systemic veins and connections**

vi. **septal defects**

vii. **valvular lesions** (including atresia)

viii. **pulmonary arteries** (systemic pulmonary collaterals)

ix. aorta

x. others

RV and LV Volumes with and without indexing to body surface area

** PA and Aortic Dimensions** (diameters)

i. MPA

ii. LPA

iii. Coarctation (minimum)

iv. Shunt or conduit (minimum and maximum)

Blood Flow, Velocity

a. **Pulmonary/systemic flow ratio**

b. Valve (if regurgitant) (name of valve)

i. **forward flow**

ii. **regurgitant flow**

iii. **regurgitant fraction**

c. Valve (if stenotic) (name of valve)

i. **peak velocity (gradient)**

ii. other

d. Coarctation

i. **peak velocity (gradient)**

ii. **collateral flow estimate**

e. Pulmonary arterial flow

i. **MPA**

ii. **LPA**

iii. **RPA**

f. Shunt or Conduit Flow (name of shunt or conduit)

i. **flow**

ii. **peak velocity (conduit)**

8) The SCMR recognizes that more extensive historical information may be desired by certain institutions that perform CMR. At the time of this publication, SCMR considers this information *optional *for inclusion in the final report. Accordingly, in the case in which further data are desired, the following outline for data collection is provided:

a) **Relevant Medications**

• Aspirin

• Warfarin

• Other anti-platelet

• Beta-blocker

• ACE inhibitor

• Angiotensin receptor blocker

• Nitrate

• Statin

• Erectile dysfunction medication

• Calcium channel blocker

b) History and risk factors

1) **Hypertension**

2) **Dyslipidemia** – Indicate if the patient has a history of dyslipidemia, diagnosed or treated by a physician or documentation of a total cholesterol >200 or an LDL ≥130 or an HDL < 30.

3) **Is the LDL > 100 mg/dl or 2.59 mmol/l?** Yes/No

4) **Tobacco use**: Current, Former, Never

5) **Diabetes** – Yes/No

c) **Peripheral arterial disease**

1) Claudication with exertion

2) Amputation for arterial vascular insufficiency

3) Aorta or iliac occlusive disease reconstruction

4) Peripheral vascular bypass surgery or percutaneous intervention

5) Documented AAA repair or stent

d) **Other cerebrovascular diseases**

1) Cerebrovascular accident

2) RIND

3) TIA

4) Carotid test >75% occlusion

5) Prior carotid surgery

e) **Arrhythmias**

1) Atrial fibrillation

2) Frequent PVC's

3) History of ventricular tachycardia

4) History of ventricular fibrillation

f) **Heart failure**

1) **Previous history**: Yes/No

2) **NYHA Class Heart failure**: Class I/Class II/Class III/Class IV

g) **Presence of angina**: None, typical angina, atypical angina, non-anginal chest pain

h) **Characteristics of chest pain or suspected angina equivalent: **Substernal chest pain, provoked by exertion, or relieved by rest &/or nitroglycerin

i) **Ability to exercise prior to testing (METS)**

j) **Previous noninvasive cardiovascular imaging tests**

• Echocardiography

• Nuclear myocardial scintigraphy

• Cardiovascular computed tomography

• Cardiovascular magnetic resonance

• Cardiac catheterization

• None

j) **Surgical risk**

• Low risk surgery

• Intermediate risk surgery

• High risk surgery

k) For studies incorporating CV stress, prior to the procedure, the following information should be verified:

• **Prior MI**

• **Prior coronary revascularization (PCI and/or CABG)**

• **Pretest Probability of CAD (none, low, medium, high)**

• **Is the ECG interpretable for ischema? Yes/No**

• **Framingham Risk Score**

• **Estimate of CAD risk **(<10%, 10–20%, >20% over 10 years

9. Noncardiovascular Findings

It is recognized that there may be findings unrelated to the cardiovascular system identified during CMR imaging procedures. Such findings should be reported in accordance with local standards. However, SCMR recognizes that the contrast, resolution and field of view of a CMR study are optimized for the cardiovascular system rather than to assess for abnormalities outside of the cardiovascular system.

10. Summary and Conclusions

SCMR recommends that each report conclude with appropriate statements that relate the study indications to the imaging acquisition and findings associated with performance of the study. SCMR recommends that these statements provide referring physicians with **conclusions **that allow the prescription of therapy based on the study findings. SCMR recommends that the conclusion of the report provide the written or electronic **signature **of the individual accomplishing the report along with the **time and date of the signature**. SCMR considers it optional to provide the National Provider Identifier for the physician signing the report.

## Principles of disseminating the final report

1) The final signed report is considered to be the definitive means of communicating to the referring physician or other relevant health care provider. Other methods of rapid communication are encouraged in certain situations, such as critical findings, unexpected abnormal findings, or findings that may immediately alter the patient's course of treatment.

2) The report should be reviewed to minimize interpretive, descriptive, or transcription errors prior transmitting the final results.

3) The final report should be completed in accordance with governmental or health care facility medical records regulations.

4) The signed written report should be immediately transmitted to the referring physician or health care provider who is treating the patient once it has been finalized and in accordance with appropriate governmental requirements.

5) When feasible, a copy of relevant key images should accompany the final report.

6) A copy of the final report should be archived at the imaging facility as part of the patient's medical record and be retrievable for future reference. Retention and distribution of these records should be in accordance with governmental regulations and facility policies.

## Communications other than the final report

SCMR strongly encourages the rapid dissemination of a finalized report. It is recognized however, that preliminary reports may be necessary in certain situations. Preliminary reports should be identified as such; however, it is recognized by SCMR that their accuracy may be compromised.

If a change is made by a disseminated preliminary discrepant from the final interpretation, then written documentation and communication to all treating or referring physicians is indicated.

It is recommended that any methods of such communications be included in the final report such that documentation is complete.

### *Self-referred and Third Party Referred Patients*

The SCMR recognizes that some individuals may seek imaging studies as part of a self-referral or referred by a third party, such as an insurer or an employer.

#### *Self-referred patients*

Imagers should recognize that performing imaging studies on self-referred patients establishes a doctor patient relationship that includes responsibility for communicating the results of imaging studies directly to the patient and arranging for appropriate followup.

#### *Third Party Referred Patients*

Patients may be referred for imaging studies by insurance companies, employers, research studies, other benefit programs, or in some instances, attorneys. In such cases, the reports of these studies are frequently communicated through their requesting entity to a clinician or directly to the third party designated clinician. The results of these examinations are then communicated to the patient directly. Regardless of the source of the referral, the diagnostic imager has an ethical responsibility to insure communication of unexpected or serious findings to the patients. It is suggested that each imaging organization that desires to scan and generate reports on self referred patients develop communication policies within their centers to address evolving issues in this arena.

## Abbreviations

AAA: Abdominal Aortic Aneurism; ACC: American College of Cardiology; AHA: American Heart Association; ARVC: Arrhythmogenic Right Ventricular Cardiomyopathy; CABG: Coronary Artery Bypass Graft; CAD: Coronary Artery Disease; CMR: Cardiovascular Magnetic Resonance; CV: Cardiovascular; ECG: Electrocardiogram; EDV: End Diastolic Volume; EF: Ejection Fraction; ESV: End Systolic Volume; FGRE: Fast Field Gradient Echo; GFR: Glomerular Filtration Rate; HDL: High Density Lipoprotein; IH: Intramural Hematoma; LDL: Low Density Lipoprotein; LGE: Late Gadolinium Enhancement; LPA: Left Pulmonary Artery; LV: Left Ventricular; MI: Myocardial Infarction; MPA: Main Pulmonary Artery; MR: Magnetic Resonance; MVO: Microvascular Obstruction; NPI: National Provider Identifier; NYHA: New York Heart Association; PA: Pulmonary Artery; PCI: Percutaneous Coronary Intervention; PC-MR: Phase-Contrast Magnetic Resonance; PV: Pulmonary Vein; PVC: Premature Ventricular Contraction; RIND: Reverse Ischemic Neurologic Deficit; RPA: Right Pulmonary Artery; RV: Right Ventricular; RVEF: Global Right Ventricular Performance; SCMR: Society for Cardiovascular Magnetic Resonance; SD: Standard Deviation; SMA: Superior Mesenteric Artery; SPAMM: Spatially Modulated Magnetization; SSFP: Steady State Free Procession; SV: Stroke Volume; T_1 _W MRA: T_1 _Weighted Magnetic Resonance Angiography; TIA: Transient Ischemic Attack; Venc: Velocity Encoding.

## Competing interests

Dr. Christopher Kramer serves as a consultant and receives research support from Siemens. Dr. Michael McConnell receives research support from GE Healthcare. Dr. Eike Nagel receives research support from Philips Healthcare, and Bayer Schering Pharma. Dr. Nagel is a speaker for General Electric, and Bayer Schering Pharma. Dr. Nagel also serves as a consultant for General Electric and Bayer Schering Pharma. Dr. Subha Raman receives research support from Siemens. No other authors had any competing interests.

## Authors' contributions

WGH, DB, JGB, MGF, MAL, MVM, SVR, CBH and ACR researched, wrote, reviewed, and revised the document. SF, CBH, CMK, EN and SN reviewed the document for content.

## Supplementary Material

Additional file 1 **Table S1.** Recommended items for inclusion in Final Report. Click here for file

Additional file 2 **Table S2**. Optional items for inclusion in the final report. Click here for file
