# Supplementary material for: Society for Cardiovascular Magnetic Resonance guidelines for reporting cardiovascular magnetic resonance examinations
Source: J Cardiovasc Magn Reson. 2009 Mar 3;11(1):5. doi: 10.1186/1532-429X-11-5 (PMC2662831; doi:10.1186/1532-429X-11-5)
Supplement: Additional file 1 — Table S1. Recommended items for inclusion in Final Report. [file 1532-429X-11-5-S1.doc]

Table S1. Recommended items for inclusion in Final Report.

Administrative

Site ID

Site of Service

Scanner Type

Accreditation status

Accreditation entity

Demographics

Unique Patient ID

Patient Date of Birth

Patient Gender

Patient Race/Ethnicity

Scheduling and Performance of Study

Date of Procedure

Time of Procedure

Personnel involved in procedure

Primary indication for test

Study quality

Listing of sequences used

Historical Information

Height

Weight

Cardiovascular non-imaging findings

Electrocardiogram (ECG) interpretation (when acquired)

Heart rate and rhythm (as indicated)

Systolic blood pressure (as indicated)

Diastolic blood pressure (as indicated)

Oxygen saturation (as indicated)

Predictive heart rate response for age (as indicated)

Agent, quantity, duration, route of administration of the agents and associated medications

(when used)

Agent, type, name, route, site, and speed of administration of contrast (when used)

Type, route and measures of administration of these agents or support, cardiovascular and

pulmonary responses, reason for administration of anesthetics (when used)

Indication specific items

1. Aorta

Aortic annulus dimension

Sinus of valsalva dimension

Sinotubular junction dimension

Ascending and descending aorta diameters

Sinotubular effacement (when present)

Tortuosity (when present)

Aortic atherosclerosis (when present)

Aortic aneurysm (when present)

Morphology

Location

Relation to branch vessels

Presence of mural thrombus

Visceral compressive effects (effacement expansion of the aorta against

surrounding structures),

Post-contrast appearance

Periaortic fluid

Mediastinal fluid

Pericardial fluid

Pleural fluid

Aortic dissection (when present)

Dissection classification

Intimal flap

Location of tear or areas of communication

Size and extent of the true and false lumens

Murmal thrombus or blood in false lumen

Branch vessel involvement

Periaortic fluid

Mediastinal fluid

Pericardial fluid

Pleural fluid

Intramural hematoma (IH)

Penetrating ulcer

Inflammatory diseases

Aortic wall thickness

Multispectral appearance on different pulse sequences

Contrast enhancement pattern

Branch vessel involvement

Periaortic fluid

Pleural fluid

Pericardial fluid

2. Peripheral arterial disease

Vessel location and orientation

Stenosis severity should be reported in 25% increments

3. Cardiac size and function

Left ventricular volumes

Left ventricular dimensions

Left ventricular ejection fraction

Left ventricular regional wall motion

4. Cardiac Stress testing

Left ventricular wall function

Left ventricular wall motion score index

Transmurality and persistence of perfusion defects (if acquired)

Late gadolinium enhancement (LGE) (if acquired)

Microvascular obstruction (MVO) (if acquired)

5. Cardiomyopathy and inflammation

Left ventricular volumes

Presence and extent of T2 signal intensity

Presence and extent of late gadolinium enhancement (if acquired)

Presence of pericardial effusion

T2* (when acquired in myocardial iron assessments)

6. Coronary artery segments

Origin and course of coronary artery

Length of visualized segments

Intramural

Patency of bypass conduits

7. Valvular heart disease

Morphology of valves

Insufficiency or valvular excursion

Identification of stenosis or regurgitant lesions

Velocity Encoding (Venc) setting (when flow measured)

Peak velocity (when flow measured)

Transvalvular gradient (when flow measured)

Regurgitant volume and fraction (when flow measured)

Heart rate

Valve area

Ventricular dimensions and volumes

8. Arrhythmogenic right ventricular cardiomyopathy

Global right ventricular performance (RVEF)

Right ventricular dilation (when present)

Regional right ventricular wall motion

Fatty infiltration of the right ventricle (when present)

Fibrosis identified with late gadolinium enhancement (when present)

9. Cardiac and para cardiac masses

Myocardial mass description

Myocardial function

Involvement of the pericardium

10. Pericardial description

Morphology and description

Left ventricular volumes and ejection fraction

Ventricular wall motion

Systolic wall motion

+/- Abnormal septal motion

Atrial inversion

Late gadolinium enhancement (when acquired)

11. Pulmonary vein assessments

Number

Atrial side of return

Accessory or anomalous veins (if present)

Stenosis

Maximum ostial diameters (register cardiac phase and imaging technique

used during assessment)

Minimal ostial diameter

12. Congenital heart disease

Morphology for simple and complex lesions

Situs

Ventriculoarterial relationship

Atrioventricular relationship

Pulmonary venous connection

Systemic veins and connections

Septal defects

Valvular lesions (including atresia)

Pulmonary arteries

Right and left ventricular volumes

Pulmonary artery and aorta dimensions

Blood flow velocities and measurements

Pulmonary/systemic flow ratio

Valve (if regurgitant) (name of valve)

Forward flow

Regurgitant flow

Regurgitant fraction

Valve (if stenotic) (name of valve)

      Peak velocity (gradient)

      Coarctation

Peak velocity (gradient)

Collateral flow estimate

Pulmonary arterial flow

Main Pulmonary Artery (MPA)

      Left Pulmonary Artery (LPA)

Right Pulmonary Artery (RPA)

Shunt or Conduit Flow (name of shunt or conduit)

Flow

     Peak velocity (conduit)

Noncardiovascular findings

Report in accordance with local guidelines for facility performing procedure

Summary and Conclusions

Statement(s) relating imaging findings to study indication

Concluding statements

Signature of interpreting physician (electronic when appropriate)

Date and time of signature of interpreting physician
