# Supplementary material for: Society for Cardiovascular Magnetic Resonance guidelines for reporting cardiovascular magnetic resonance examinations
Source: J Cardiovasc Magn Reson. 2009 Mar 3;11(1):5. doi: 10.1186/1532-429X-11-5 (PMC2662831; doi:10.1186/1532-429X-11-5)
Supplement: Additional file 2 — Table S2. Optional items for inclusion in the final report. [file 1532-429X-11-5-S2.doc]

Table S2. Optional items for inclusion in the final report.

Study referral data

Physician National Provider Identifier (NPI)

Physician subspecialty

Historical information

Serum creatinine; Estimated glomerular filtration rate (GFR) (for gadolinium contrast studies)

Relevant Medications

Hypertension (Yes/No)

Dyslipidemia (Yes/No)

Is the LDL >100mg/dl or 2.59mmol/l?

Tobacco use

Diabetes (Yes/No)

Peripheral arterial disease

Cerebrovascular diseases

Arrhythmias

Heart failure

Previous history

NYHA Class Heart failure

Presence of angina

Characteristics of chest pain or suspected angina equivalent

Ability to exercise prior to testing

Previous noninvasive cardiovascular imaging tests

Surgical risk

Prior Myocardial Infarction (MI)

Prior coronary revascularization

Pretest Probability of CAD (none, low, medium, high)

Is the electrocardiogram (ECG) interpretable for ischemia? Yes/No

Framingham Risk Score

Estimate of coronary artery disease (CAD) risk

Indication specific items

1. Aorta

Right or left-sided orientation

Aortic flow

2. Peripheral arterial disease

Flow and vascular stiffness measurements

3. Cardiac size and function

Right ventricular and right and left atrial chamber size and volumes

Left ventricular end diastolic wall thickness

Right ventricular free wall end diastolic thickness

4. Cardiac stress testing

“Bull’s Eye” 17-segment figure for wall motion, perfusion and extent of late gadolinium enhancement

5. Cardiomyopathy

Early enhancement ratio

Summary and Conclusions

National Provider Identifier for physician providing report.
